# YOLOv8-MPEB small target detection algorithm based on UAV images

**DOI:** 10.1016/j.heliyon.2024.e29501

**Published:** 2024-04-15

**Authors:** Wenyuan Xu, Chuang Cui, Yongcheng Ji, Xiang Li, Shuai Li

**Affiliations:** School of Civil Engineering and Transportation, Northeast Forestry University, Harbin 150040, China

**Keywords:** YOLOv8, MobileNetV3, Attention mechanism, BiFPN, Small target detection

## Abstract

Target detection in Unmanned Aerial Vehicle (UAV) aerial images has gained significance within UAV application scenarios. However, UAV aerial images present challenges, including large-scale changes, small target sizes, complex scenes, and variable external factors, resulting in missed or false detections. This study proposes an algorithm for small target detection in UAV images based on an enhanced YOLOv8 model termed YOLOv8-MPEB. Firstly, the Cross Stage Partial Darknet53 (CSPDarknet53) backbone network is substituted with the lightweight MobileNetV3 backbone network, consequently reducing model parameters and computational complexity, while also enhancing inference speed. Secondly, a dedicated small target detection layer is intricately designed to optimize feature extraction for multi-scale targets. Thirdly, the integration of the Efficient Multi-Scale Attention (EMA) mechanism within the Convolution to Feature (C2f) module aims to enhance the extraction of vital features and suppress superfluous ones. Lastly, the utilization of a bidirectional feature pyramid network (BiFPN) in the Neck segment serves to ameliorate detection errors stemming from scale variations and complex scenes, thereby augmenting model generalization. The study provides a thorough examination by conducting ablation experiments and comparing the results with alternative algorithms to substantiate the enhanced effectiveness of the proposed algorithm, with a particular focus on detection performance. The experimental outcomes illustrate that with a parameter count of 7.39 M and a model size of 14.5 MB, the algorithm attains a mean Average Precision (mAP) of 91.9 % on the custom-made helmet and reflective clothing dataset. In comparison to standard YOLOv8 models, this algorithm elevates average accuracy by 2.2 percentage points, reduces model parameters by 34 %, and diminishes model size by 32 %. It outperforms other prevalent detection algorithms in terms of accuracy and speed.

## 1 Introduction

Road reconstruction, expansion, and significant repair projects must reasonably safeguard road access. Many projects are half construction and half open to traffic, with considerable safety risks and hidden dangers on site and in the surrounding environment. Operators work in high-risk areas for long periods, and wearing helmets and reflective clothing can help prevent safety accidents. However, due to weak safety awareness, staff may need to pay more attention to safety hazards and remove helmets and reflective clothing, leading to frequent safety accidents. Traditional safety inspection relies mainly on manual and monitoring equipment, which makes it unable to achieve full coverage and real-time monitoring. With the rapid development of UAV technology and computer vision [1], UAVs equipped with deep learning techniques are increasingly used in applications such as climate change monitoring, search and rescue assistance, and construction industry maintenance [[2], [3], [4]]. However, variable UAV aerial photography height and complex construction environments pose challenges for UAV visual target detection, including significant image scale changes, small target sizes, complex scenes, and variable external factors.

At present, target detection algorithms based on deep learning are mainly divided into two categories: one is a two-stage detection algorithm that generates candidate regions for images using a regional convolutional neural network, extracts image feature information, and then completes classification; typical representatives are Region-based Convolution Neural Network (RCNN) [5], Fast RCNN [6], and Faster RCNN [7]. The other category is single-stage detection algorithms that directly predict the category and location of objects after deep learning; typical representatives are the You Only Look Once (YOLO) series [[8], [9], [10]] and Single Shot Multibox Detector (SSD) [11]. The single-stage detection algorithm is more straightforward and faster than the two-stage detection algorithm. It has a smaller model that can meet the requirements of practical applications regarding real-time performance.

To address the problem of helmet and reflective clothing detection. Zhang et al. [12] proposed a lightweight improvement algorithm based on YOLOv5s. They replaced the Concentrated-Comprehensive Convolution (C3) module in the backbone network and the neck layer with the Ghost module and C3CBAM, respectively. It significantly reduced the model's parameters and computational volume. In the same period, Xie et al. [13] proposed a reflective clothing and helmet detection algorithm based on CT-YOLOX. They enhanced the model's classification accuracy and robustness by introducing a Channel Attention Module (CAM) module, designing a TBCA module, and adopting a Varifocal loss function.

Bai et al. [14] utilized an improved Deep Simple Online and Realtime Tracking (DeepSORT) multi-target tracking algorithm to reduce omissions caused by occlusion and address target occlusion and scale change issues. They fused a Transformer module into the backbone network to enhance small target feature learning. They applied a BiFPN to adapt to target scale changes from photographic distance [15]. Meanwhile, Shen et al. [16] introduced the deformable convolutional C2f (DCN_C2f) module based on YOLOv8 for adaptive network field adjustment. They also designed a lightweight self-calibrating Shuffle Attention (SC_SA) module for spatial and channel attention, improving multi-scale and small target feature representation. Detection accuracy was better than other mainstream models. Zhang et al. [17] proposed a small target detection algorithm based on YOLOv7-tiny with ConvMixer detection head for UAV aerial images to improve accuracy and speed. It utilizes deep and point-wise convolution in ConvMixer to find spatial and channel relationships in passed feature information, improving minor target handling.

For addressing issues of densely distributed small targets and complex backgrounds in UAV images, along with potential misdetection and leakage, Deng et al. [18] utilized GsConv convolution for enhanced feature fusion and introduced a coordinate attention mechanism to expedite model convergence. They also switched to the Expected Intersection over Union (EIOU) loss function for optimizing edge prediction. This approach resolved misdetection and leakage problems of the helmet detection model for overlapping, small targets in complex environments. A multiscale channel-space attention (MCSA) mechanism was presented by Wang et al. to improve the detection of small-scale targets and to increase attention to the target region [19]. Li et al. [20] proposed a multi-scale dynamic feature-weighted fusion network comprising a feature map attention generator and a dynamic weight learning module. It adaptively regulates learning important target features at different scales, reducing underdetection. A pyramid self-attention module (PSAM) is also designed to enhance the network's ability to discriminate similar targets, mitigating false detections. Compared to the YOLOv5s algorithm, accuracy improves by 5.59 percentage points. Subsequently, Cheng et al. [21] presented an improved target detection algorithm for YOLOv8. The network boosts small target detection accuracy by introducing multi-scale attention and a dynamic non-monotonic focusing mechanism, enhancing the C2f module, and switching to the WIoU Loss function. A lightweight Bi-YOLOv8 feature pyramid network structure is proposed to enhance model multi-scale feature fusion. Compared to YOLOv8s, mAP50 improves by 1.5 % while parameter count reduces by 42 %.

To address the poor monitoring effect in UAV aerial images under dense, fuzzy, uneven lighting conditions, Liu et al. [22] proposed a feature-enhanced detection algorithm, CBSSD, based on a single-shot multi-box detector. It utilizes residual structure in ResNet50 to obtain low-level features, fusing these into the backbone network via feature fusion. Liao et al. [23] suggest a novel pixel neighborhood method for image recovery.

Although the above methods improve helmet and reflective clothing detection accuracy to some extent, several issues remain:

(1) The algorithms are complex and computationally demanding.
(2) Most algorithms only detect helmets, ignoring reflective clothing, limiting application scope.
(3) Current methods ineffectively balance detection and real-time performance. On the one hand, they increase model complexity for optimal detection performance. On the other, lightweight detection has remained relatively high.

Based on the above analysis, this paper proposes a small target detection algorithm for UAV images based on an improved YOLOv8.

(1) The lightweight network MobileNetv3 is utilized as the feature extraction network, reducing model parameters and computation for convenient subsequent deployment to mobile terminals and embedded devices.
(2) To improve the accuracy of small target detection, the EMA attention mechanism is incorporated into the C2f module, and multi-scale features are fused using a weighted BiFPN.
(3) An additional small target detection layer and head are designed to address complex recognition due to drastic UAV image scale changes.

## 2 Related work

It is possible to define minor goals as absolute or relative. The relative definition of a small target, as defined by the International Society for Optical Engineering (SPIE), is one that has an area of less than 80 pixels in a 256 × 256 image. Conversely, the precise meaning of small targets differs depending on the dataset; for instance, the MS COCO dataset classifies targets as small if their resolution is less than 32 pixels by 32 pixels. With low resolution, few features, target clustering, few anchor frame matches, etc., detecting small targets has always been a difficult task in target detection. However, in recent years, a number of helpful techniques have been developed to enhance the performance of small target detection.

Many researchers have improved and researched the application of attention mechanism in small target detection, aiming at the challenge of small targets. A number of studies have concentrated on improving the feature representation of small targets by introducing attentional mechanisms into backbone networks. For instance, Wang et al. [24] proposed two new detection scales based on the feature-processing module Focal FasterNet block (FFNB), which fully integrates shallow and deep features, and introduced the BiFormer attention mechanism to optimize the backbone network, which enhances the model's focus on important information. Tan et al. [25] generated distinct attention feature maps for each subspace of the feature map for multi-scale feature representation using the Ultra-Lightweight Quantum Spatial Attention Mechanism (ULSAM). In order to acquire and transmit richer and more discriminative small target features, other researchers have made adjustments to the downsampling multiplier. Additionally, for small targets, the k-means++ clustering algorithm is employed to produce more precise anchor frame sizes [26].

There are numerous additional works. For instance, Yuan et al. [27] proposed CFINet, a two-stage framework for small target detection that is based on feature imitation learning and coarse and fine pipelines. This framework helps to address the issue of a limited sample pool for optimization because there is little overlap between the prior and target regions for small targets. For driving and flying scenarios, Cheng et al. [28] created two large-scale small target detection datasets called SODA (SODA-D and SODA-A). It supports SOD development and offers a benchmark for evaluating small target detection models.

## 3 Methodology

### 3.1 YOLOv8 algorithm principles

**Fig. 1:**
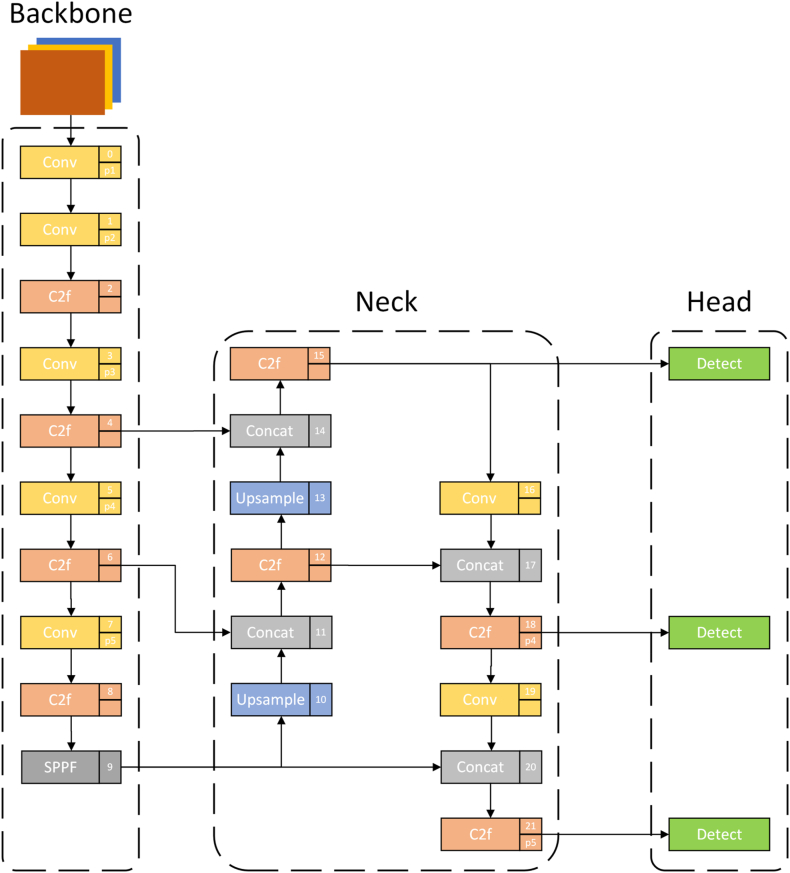
YOLOv8 network architecture. a) CSPDarknet53 network used by Backbone; b) FPN + PAN pyramid structure used by Neck; c) decoupled header structure used by Head.

The YOLO series excels in balancing speed and accuracy among various target detection algorithms. They accurately and rapidly recognize targets, are easy to deploy on diverse mobile devices, and enable real-time applications. YOLOv8 is Ultralytics' latest YOLO object recognition and image segmentation model, introducing new features and improvements to enhance performance and flexibility. The YOLOv8 network structure is shown in Fig. 1.

**Fig. 2:**
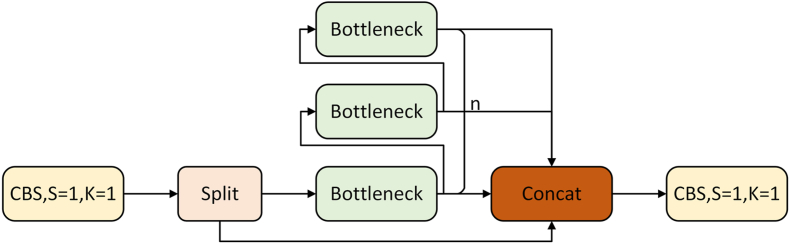
C2f module.
(3) The multi-feature fusion adopts the FPN + PAN structure to enhance multi-scale semantic expression and localization.
(4) The prediction output is based on prior features for target category and location recognition formation of the detected target and makes recognition. The current mainstream decoupled head structure (Decoupled Head) is adopted to effectively reduce the number of parameters and computational complexity while enhancing the model's generalization ability and robustness. At the same time, the previous YOLO series' use of anchor nodes (Anchor-Base) is abandoned in favor of an anchor-free approach (Anchor-Free). This direct prediction of the target's center point and width-to-height ratio reduces the number of anchor frames. The Loss computational aspect uses the Task-Aligned Assigner dynamic sample allocation strategy [29], which can be adjusted according to the training loss or other metrics. It is better adapted to different datasets and models. Distribution focal loss (DFL) combined with Complete Intersection over Union Loss (CIoU Loss) is also introduced for the regression branch loss function, with Binary Cross Entropy (BCE) used for classification loss. This results in high alignment consistency between classification and regression tasks.

The YOLOv8 model comprises four parts: Input, Backbone, Neck, and Head. These serve as input image, feature extraction, multi-feature fusion, and prediction output:

(1) The input images were enhanced using the Mosaic data enhancement method to improve the model's generalizability and robustness.
(2) The feature extraction network incorporates multiple Conv, C2f modules, and spatial pyramid pooling with features (SPPF). The C2f module leverages the strengths of C3 and Efficient Layer Aggregation Network (ELAN) in YOLOv7 by linking across more branch layers for richer gradient flow information while remaining lightweight, as shown in Fig. 2. SPPF is based on spatial pyramid pooling (SPP) to reduce network layers and eliminate redundancy for faster feature fusion.

The structure of this section is as follows: Section 3.2 provides a detailed introduction to replacing the backbone network with MobileNetV3. Section 3.3 describes the strategy of improving feature extraction in the neck and introducing attention mechanisms. In Section 3.4, we discuss the work of adding a small object detection layer. Finally, Section 3.5 summarizes the structure of the improved YOLOv8.

### 3.2 Backbone network

Fewer parameters, less computation, and shorter inference times than heavyweight networks characterize lightweight networks. They are more suitable for scenarios where storage space and power consumption are limited, such as edge computing devices like mobile embedded devices. MobileNetV3 [30] is a lightweight network model proposed by the Google team. It has achieved excellent performance in lightweight image classification, target detection, semantic segmentation, and other tasks. The MobileNetV3 parameters are obtained by network architecture search (NAS) [31], inheriting some practical results from V1 [32] and V2 [33]. MobileNetV3 also invokes the Squeeze-and-Excitation (SE) channel attention mechanism [34], redesigning the time-consuming layer structure. These improvements further enhance the network's performance.

**Fig. 3:**
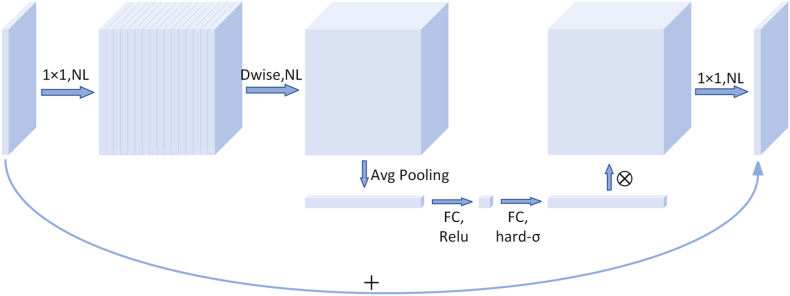
MobilenetV3 block structure diagram.

As shown in Fig. 3, the input image is first padded by 1 × 1 convolution to increase the number of channels. Next, deep convolution is applied in a high-dimensional space, and the resulting feature map is optimized using the SE attention mechanism. The number of channels is then reduced using 1 × 1 convolution (linear activation function). Residual linking is used when the step size is 1, and the input and output feature shapes are equal. The downsampled feature map is output directly when the step size is 2 (downsampling stage).

**Fig. 4:**
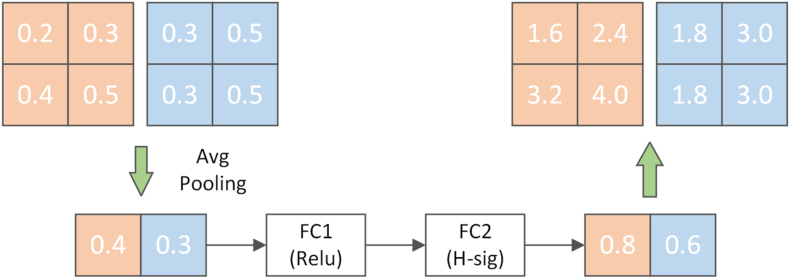
Se attention mechanism.

The attention mechanism first performs global average pooling [35] on the feature graph, as shown in Fig. 4. The relationship between the number of channels in the feature map and the pooling result (one-dimensional vector) is [h, w, c] = => [None, c]. Afterward, the output vector is obtained through two fully connected layers. The number of output channels in the first fully connected layer is 1/4 the number in the original input feature map. The number of output channels in the second fully connected layer is the same as in the original input feature map. That is, the dimension is first reduced and then increased. The output vector of the fully connected layer may be considered each vector element representing a weight relationship derived from the analysis of each feature map. More essential feature maps are given greater weights, i.e., their vector elements have more significant values. On the contrary, less important feature maps correspond to smaller weight values. The first fully connected layer uses the Rectified Linear Unit (ReLU) activation function [36], and the second fully connected layer uses the hard_sigmoid activation function [37]. After two fully connected layers, a vector of channel elements is obtained, each element being a weight for each channel. Multiplying the weights with their original feature map counterparts gives the new feature map data.

### 3.3 Neck structure

#### 3.3.1 Bi-directional feature pyramid network

**Fig. 5:**
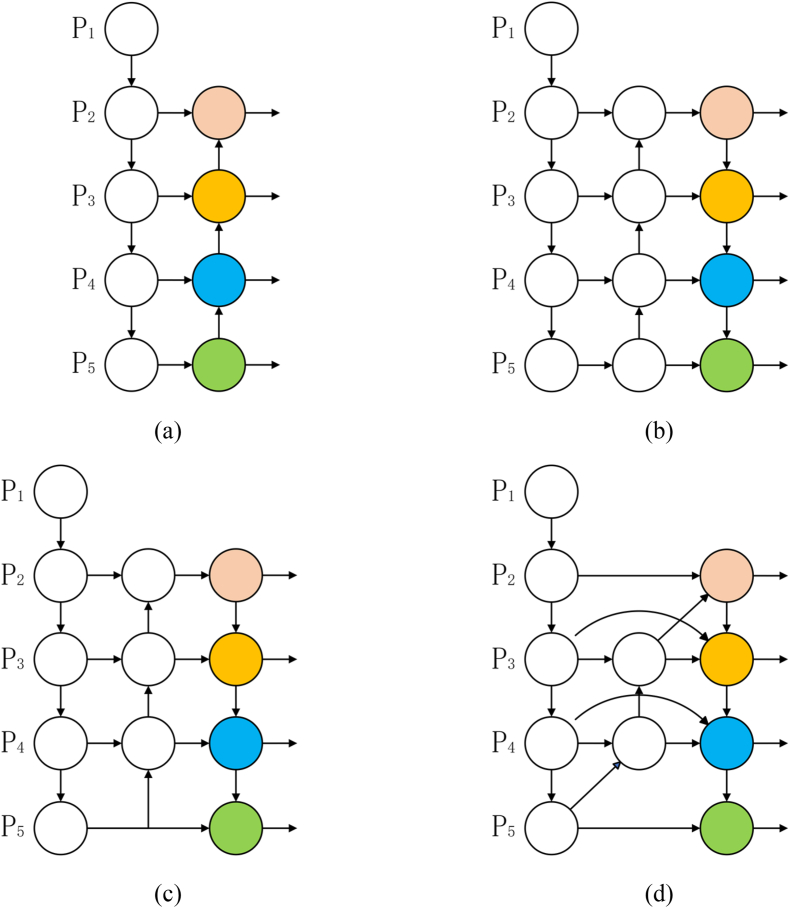
Feature network design. (a)FPN; (b)PAN; (c)YOLOv8; (d)BiFPN. Pink circles represent micro and small target detectors, orange circles represent small target detectors, blue circles represent medium target detectors, and green circles represent large target detectors. (For interpretation of the references to color in this figure legend, the reader is referred to the Web version of this article.)

Fig. 5(a) introduces the feature pyramid network (FPN) [38], which enhances the detector's ability to detect targets at different scales. This is achieved by introducing a bottom-up path that fuses multi-scale features from levels 2 to 5(P2–P5). However, it is computationally intensive, requiring long training and inference times, and is limited to unidirectional information flow. To solve this problem, instead of relying solely on the FPN, path aggregation network (PAN) [39] incorporates an additional top-down path aggregation network. It helps preserve detailed information in low-resolution feature maps, enhancing detection accuracy. However, it also increases computation, as shown in Fig. 5(b). Fig. 5(c) YOLOv8 borrows from PAN, simplifying the network to improve detection speed. YOLOv8 optimizes the feature pyramid network and removes nodes without feature fusion. However, all feature fusion methods have weak localization and recognition of small targets. This is because small targets are easily affected by normal-sized targets during feature extraction, and the network deletes inconspicuous information. Therefore, small target information is continuously reduced, resulting in unsatisfactory small target detection. BiFPN [40] introduces learnable weights to learn the importance of different input features while iteratively applying bottom-up and top-down multi-scale feature fusion. Introducing a bidirectional flow of feature information solves the problem of information loss and excess when extracting features at different scales. BiFPN fuses top- and bottom-sampled feature maps layer by layer and simultaneously introduces horizontal and vertical connections to fuse and exploit features better at different scales. It thus has strong robustness in handling complex scenes like scale change and occlusion, as shown in Fig. 5(d).

#### 3.3.2 Attentional mechanisms

**Fig. 6:**
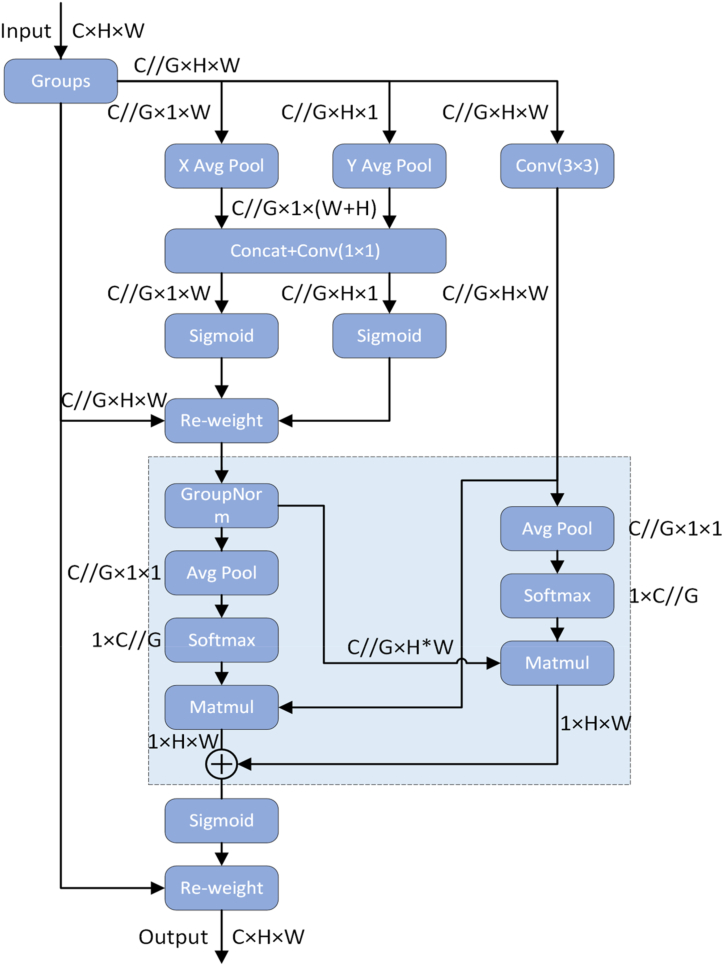
EMA structure.

EMA [41] is an efficient multiscale attention mechanism. It preserves information and reduces computational cost without reducing channel dimensionality. As shown in Fig. 6, the parallel substructure avoids sequential processing, and the convolution produces efficient channel descriptions and better pixel-level attention for high-level feature maps. Specifically, a 1 × 1 convolution from the CA [42] module forms a 1 × 1 branch in the shared component. 3 × 3 kernels are placed in parallel for fast multiscale spatial structure information aggregation, forming 3 × 3 branches. This feature grouping and multiscale structure effectively establish short- and long-term dependencies for superior performance.

For any given input feature map $X\in R^{C\times H\times W}$, EMA divides the cross-channel dimension X into G sub-features for learning different semantics. Grouping styles can be defined as X = [$X_{0},X_{i}{,\ldots ,X}_{G-1}$]， $X_{i}\in R^{C//G\times H\times W}$. Setting G ≪ C and learned attention weights to enhance the feature representation of the region of interest in each sub-feature.

Large receptive fields of local neurons enable collection of spatial information at multiple scales. EMA extracts attention weight descriptors for grouped feature maps using 3 parallel paths - two in the 1 × 1 branch and one in the 3 × 3 branch. They model cross-channel information interactions in the channel direction to capture dependencies and reduce computational budget. Two ID global average pooling operations in the 1 × 1 branch encode the channel along two spatial directions. Only one 3 × 3 kernel is stacked in the 3 × 3 branch to capture multi-scale feature representations. Conventional convolution doesn't include batch coefficients in the convolution function, making the number of convolution kernels independent of the batch coefficients of the forward input. To address this, the group G should be reshaped and displaced into the batch dimension, and the input tensor should be redefined as C//G × H × W.

Similar to CA, EMA combines two coded features by image height and applies the same 1 × 1 convolution to fit the output to a two-dimensional binomial distribution using two nonlinear Sigmoid functions. For cross-channel interaction features, multiply two-channel attention maps from different paths. Expanding the feature space through 3 × 3 convolution captures local interactions and increases branching. This process encodes inter-channel information to prioritize channels and retains accurate spatial information. Additionally, an interspatial information aggregation method is utilized based on the Pyramid Split Attention (PSA) idea, with different spatial dimension directions, to achieve richer feature aggregation.

EMA introduces two tensors: one from the 1 × 1 branch and the other from the 3 × 3 branch. The 1 × 1 branch outputs are encoded with 2D global average pooling to preserve global spatial information, then transformed to the corresponding dimensions. Finally, the joint activation mechanism of the channel features is performed, i.e., $R_{1}^{1\times C//G}\times R_{3}^{C//G\times HW}$. Similarly, Prior to joint activation, the outputs of the 3 × 3 branch are encoded and converted to $R_{3}^{1\times C//G}\times R_{1}^{C//G\times HW}\text{.}$ 2D Global Pooling Operations $z_{c}=\frac{1}{H\times W}\sum_{j}^{H}\sum_{i}^{W}x_{c}\left(i,j\right)$ Encoding global information and modeling long-range dependencies. Efficient computation requires pooling the 2D global average using Softmax, a nonlinear function of the 2D Gaussian mapping. A spatial attention map is created by multiplying the output of parallel processing with the dot product matrix operation. The stage collects spatial information at various scales and encodes global spatial information in 3 × 3 branches using 2D global average pooling.

A second spatial attention map is then generated, retaining all precise spatial location information. Finally, the two spatial attention weight values are combined using a Sigmoid function to calculate output feature maps for each group. The EMA algorithm captures pairwise relationships between pixels at the pixel level and emphasizes the global context of all pixels. The final output is an X of the same size that can be easily stacked into a YOLOv8 network.

The C2f module in YOLOv8 incorporates several convolution modules [43] and residual structures [44]. The residual structure is critical for image feature extraction. Therefore, the attention mechanism EMA is utilized to improve the combination with the C2f module to form the Feature Enhancement Module (FEM). This module re-distributes the weights of extracted features, enhancing the feature expression of small targets and improving the feature extraction of the main stem, ultimately improving small target detection.

**Fig. 7:**
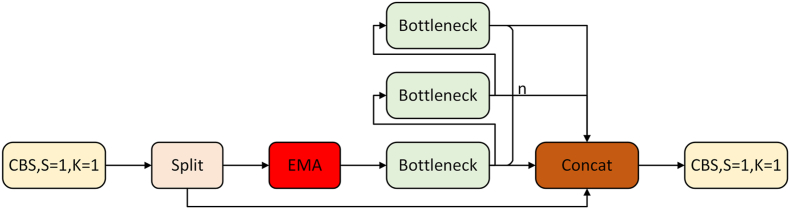
FEM structure. The EMA attention mechanism is embedded in the second residual network of the C2f module.

The paper proposes a feature enhancement module consisting of a neck-structured C2f module with the attention mechanism EMA. The C2f structure, unfolded in Fig. 2, specifies the Bottleneck module. The C2f comprises two residual network structures providing better classification function fitting for higher accuracy. Optimized for training as the network deepens, the C2f module was chosen for feature enhancement. Fig. 7 shows the feature enhancement module structure based on the C2f structure with an embedded EMA attention mechanism. The module contains two nested residual modules, extracting features more effectively by embedding the EMA module into the second residual block of the C2f. Operation is similar to C2f, with an additional attention mechanism step for weight extraction and allocation, more conducive to learning small goals. This paper introduces the attention mechanism in the first three C2f modules of the neck structure.

### 3.4 Detection head

**Fig. 8:**
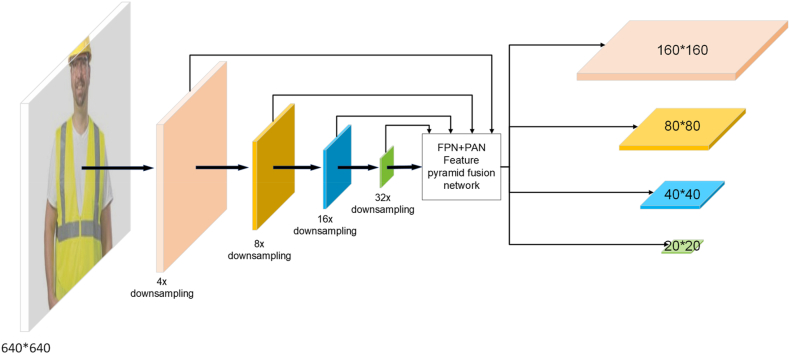
Add a small target detection layer and a P2 detection header. The original YOLOv8 network structure only includes downsampling at 8x, 16x, and 32x with corresponding output maps of 80 × 80, 40 × 40, and 20 × 20. This paper proposes the addition of 4x downsampling and 160 × 160 output maps to the original structure.

This paper adds a small target detection layer and a P2 detection head to address the problem of complex target recognition due to drastic changes in the UAV image scale. The original YOLOv8 network structure has three feature maps with different downsampling scales for detecting small, medium, and large targets. As the network depth increases, feature maps become smaller, more abstract, and contain more semantic information. Feature maps of small size are often used to detect large targets because they have a larger receptive field. On the other hand, large-scale feature maps are more accurate for locating targets and are more suitable for detecting small targets. A larger scale feature map is added to the FPN + PAN structure's neck structure to improve the network's ability to detect small targets. The optimized network structure is shown in Fig. 8.

### 3.5 Improved YOLOv8 network

**Fig. 9:**
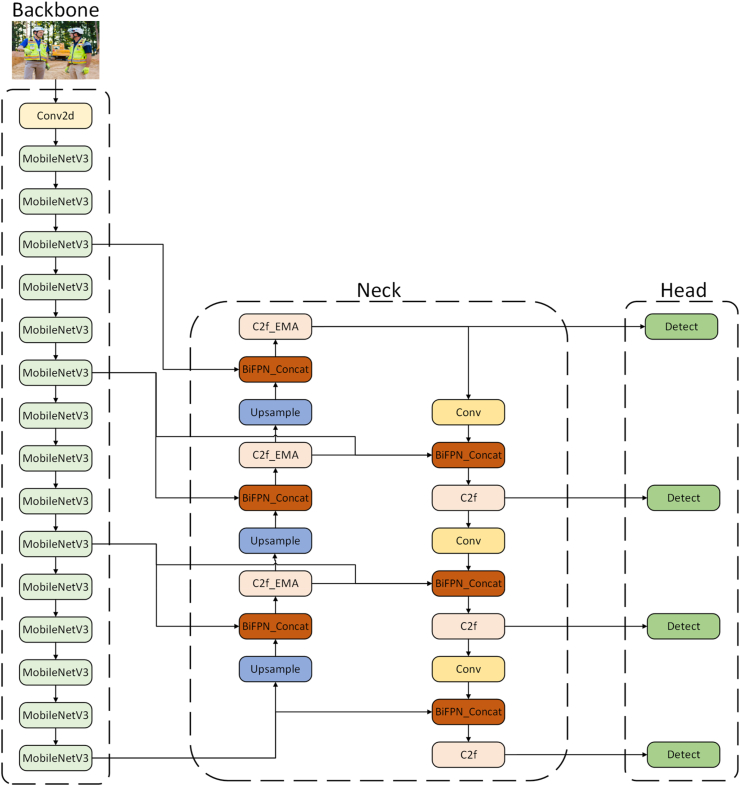
Improved YOLOv8 network structure. a) MobileNetV3 network used by Backbone. b) BiFPN framework used by Neck and added a small target detection layer. c) Head added an additional Detect.

The paper presents improvements to the YOLOv8 backbone network, neck structure, and detection head. The improved model network structure is depicted in Fig. 9.

## 4 Materials and experiments

### 4.1 Related configuration

Table 1 displays the configuration of the experimental environment used in this paper. The experiments were conducted using PyTorch 2.0.0, with results computed by the CUDA kernel. The hardware primarily comprises a high-performance computer. The mainframe computer is equipped with an Intel(R) Core(TM) i9-13900KF processor and an RTX 4090 graphics card.

**Table 1:** Experimental environment configuration.

Items		Description
Hardware	Central Processing Unit	Intel(R) Core (TM) i9-13900KF
	Random Access Memory	64 GB
	Solid State Drive	Samsung SSD 2 TB
	Graphics Card	NVIDIA GeForce RTX 4090
Software	Operating System	Windows 10, 64 bit
	Programming Language	Python 3.8
	Learning Framework	Pytorch 2.0.0

Table 2 displays the specific parameter configurations for the relevant parameters, including batch size of training samples, image size, initial learning rate (lr0), final learning rate (Irf), number of training rounds (epoch), and weight decay coefficient (weight_decay).

**Table 2:** Experimental parameter Configuration.

Parameter name	Parameter information
batch-size	32
Image-size	640 × 640
lr0	0.01
Irf	0.01
epoch	200
weight_decay	0.0005

### 4.2 Data set introduction

**Fig. 10:**
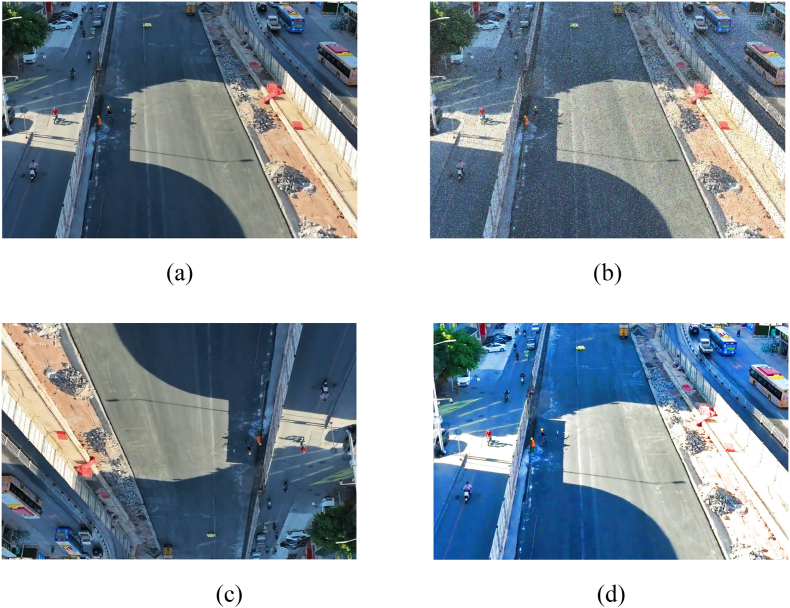
Enhancement variations. (a) Original figure; (b) adding noise; (c) random flipping; (d) enhanced brightness.

Currently, only some datasets exist on helmets and reflective clothing. The public dataset needs both helmet-wearing and reflective clothing, inadequately reflecting their varied states in real construction scenarios. Fully considering changing light conditions onsite, workers' varying postures, helmet colors, and helmet state influence, this paper targeted data collection. A total of 2672 images were collected, including dataset images, web crawling, and self-shooting. They depict road reconstruction, expansion, and significant/medium repair site workers in various postures - standing, squatting, bending - from different angles and distances. Images also show workers wearing different helmets indoors/outdoors and removing/donning helmets. In Fig. 10 a-d, noise, random flip and enhanced brightness were added to the original dataset to enhance the robustness of the model and ensure adequate training/validation. These techniques improve model generalizability. Thus, this paper presents a 6680-image dataset, enhanced data categorized into four groups: head, helmet, reflective clothing, and other clothing. The dataset is split 8:2 into training/validation sets.

### 4.3 Testing model evaluation index

To evaluate the model's performance, average precision (AP) and mean average precision (mAP) are introduced, as shown in equations (3), (4). AP is calculated using difference-average accuracy (DAA), the area under the accuracy-recall curve. Accuracy and recall are calculated using the formulas in Eqs. (1), (2):

(1) $$Precision=\frac{TP}{TP+FP}$$

(2) $$Recall=\frac{TP}{TP+FN}$$

Where T/F is true/false, indicating whether the prediction is correct or not, and P/N is positive/negative, indicating whether the prediction is positive or negative.

(3) $$AP=\int_{0}^{1}Precision\left(Recall\right)d\left(Recall\right)$$

(4) $$mAP=\frac{1}{n}\sum_{i=1}^{n}{AP}_{i}$$

Where n is the number of categories and ${AP}_{i}$ represents the AP of the ith category.

### 4.4 Comparative experiments on attention mechanisms

In the improved YOLOv8 strategy, an attention module has been added to enhance the model's detection ability. This forces the network to focus more on the target to be detected. The specific operation involves two main approaches: one is to insert the attention module in front of the final convolutional layer of the YOLOv8 model backbone network (e.g., SE, CBAM (Convolutional Block Attention Module) [45], CA, and EMA). The other is to replace the original attention module with an enhanced attention module (e.g., C2f_SE, C2f_CBAM, C2f_CA, C2f_EMA) in all CSP modules (Layer 3, Layer 5, Layer 7, and Layer 9) within the YOLOv8 backbone network. SE, CBAM, CA, EMA, C2f_SE, C2f_CBAM, C2f_CA, and C2f_EMA were trained to determine the most appropriate attention mechanism for the helmet state detection network in this study. The results are presented in Table 3. The YOLOv8 algorithm's detection performance is improved with the introduction of the attention module. C2f_EMA has the best performance under this algorithm.

**Table 3:** Comparative experiments on attentional mechanisms.

Model	Params/ $10^{6}$	GFIOPs	mAP/%
YOLOv8s	11.17	28.8	89.7
YOLOv8s-SE	**11.14**	**28.7**	89.3
YOLOv8s-CBAM	11.40	28.9	90.0
YOLOv8s-CA	11.15	**28.7**	90.6
YOLOv8s-EMA	**11.14**	**28.7**	90.8
YOLOv8s-C2f_SE	11.15	**28.7**	89.1
YOLOv8s-C2f_CBAM	11.41	28.9	90.2
YOLOv8s-C2f_CA	11.16	**28.7**	90.7
YOLOv8s-C2f_EMA	11.15	**28.7**	**90.9**

### 4.5 Ablation experiment

The effect of different module combinations on results is further explored in ablation experiments to verify the proposed network's rationality and effectiveness. All parameters remain the same in the ablation experiments except those of the added modules, including relevant hyperparameters, training strategy, and experimental environment. In this paper, the YOLOv8s module with the backbone network CSPDarknet-53, which MobileNetV3 replaced, is named YOLOv8s-M. The YOLOv8s module, with the addition of the P2 detection header, is called YOLOv8s-P. The YOLOv8s module introducing the EMA Attention Mechanism is given the name YOLOv8s-E. The YOLOv8s module using the BiFPN feature fusion network is named YOLOv8s-B.

This paper conducts ablation experiments in three ways. First, an improvement module is added to the original YOLOv8 algorithm to verify its effect on the baseline model. Second, one of the improvement methods is removed from the final improved model, YOLOv8-MPEB, to assess its impact on the final model. Lastly, two improvement modules are removed from the final improved model to verify their impact on the final model.

Analysis of ablation experiment results in Table 4 indicates: (ⅰ) YOLOv8s served as reference baseline with mAP50 89.7 % on homemade helmet and reflective clothing dataset. (ⅱ) Replacing the YOLOv8 backbone with lightweight MobileNetV3 reduces parameters, computation, and model size by 3.29 M, 9.8GFLOPs, and 6.3 MB, respectively, but sacrifices 0.6 % average accuracy. MobileNetV3 ensures fewer parameters, computation, and real-time performance, making the model more lightweight and practical. (ⅲ) Adding a P2 detector head improves mAP50 by 1.6 % and computation by 8.2 GFLOPs. Setting the P2 anchor frame to a small target reduces detection leakage from oversized anchors. Fusing multi-level information, especially shallow shape, and size, improve localization and detection of small targets. However, this increases the model's computational burden. (ⅳ) The average accuracy improved by 1.2 % with the addition of the EMA attention mechanism to the C2f module, while other metrics remained stable. It demonstrates that incorporating local contextual info around targets can enhance target features by extracting deep global contextual info and feeding back to shallow auxiliary detection for densely distributed UAV aerial images. (ⅴ) By replacing the original YOLOv8 feature pyramid network with the BiFPN bidirectional feature pyramid, the strategy achieved a 1.0 % mAP50 increase. This suggests that a bidirectional flow of feature info facilitates multi-level info interaction and better fusion and utilization of features at different scales. (ⅵ) Experimental results show that all improvement points, except MobileNetV3 backbone replacement, enhance the network's average accuracy. However, the MobileNetV3 lightweight network significantly reduces parameters, computation, and model size, making model deployment to mobile terminals and embedded devices easier. By adding a p2 detection header, incorporating EMA attention into the C2f module, and switching to the BiFPN bidirectional feature pyramid network, mAP50 reaches a maximum of 92.4 %. However, this also increases computation to 37.5 M.

**Table 4:** Results of ablation experiments.

methodologies	mAP50/%	Parameters/M	PLOPs/G	Model size/MB
YOLOv8s	89.7	11.17	28.8	21.4
YOLOv8s-M	89.1	7.88	**19.0**	15.3
YOLOv8s-P	91.3	10.64	37.0	20.6
YOLOv8s-E	90.9	11.15	28.7	21.5
YOLOv8s-B	90.7	11.20	28.9	21.6
YOLOv8s-MP	90.6	**7.38**	27.2	**14.5**
YOLOv8s-ME	90.5	7.88	19.1	**14.5**
YOLOv8s-MB	90.3	7.89	**19.0**	**14.5**
YOLOv8s-PE	91.5	10.64	37.1	20.6
YOLOv8s-PB	91.7	10.72	37.4	20.8
YOLOv8s-EB	91.0	11.21	28.9	21.6
YOLOv8s-MPE	91.2	**7.38**	27.3	**14.5**
YOLOv8s-MPB	91.3	7.39	27.2	**14.5**
YOLOv8s-MEB	90.7	7.89	19.1	15.3
YOLOv8s-PEB	**92.4**	10.72	37.5	20.8
YOLOv8s-MPEB	91.9	7.39	27.4	**14.5**

**Fig. 11:**
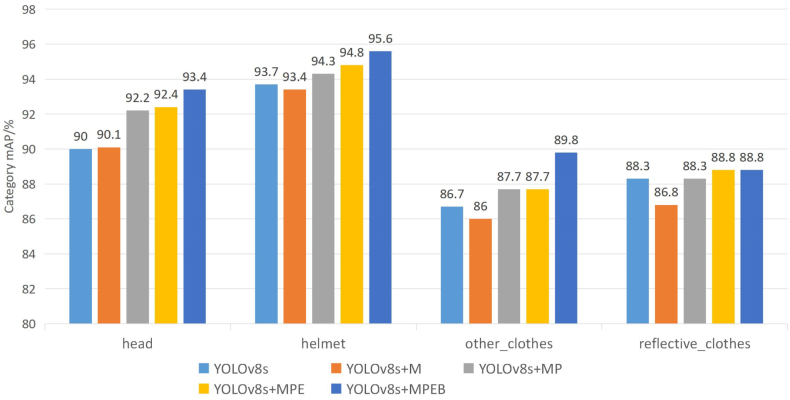
Comparison of the categories of each strategy on the homemade dataset.

Fig. 11 compares the benchmark model's experimental results on each category's improvement module. For the MobileNetV3 lightweight network module, average accuracy decreased across all categories except “not wearing a helmet (head)," which increased by 0.1 %. Adding the P2 detector head module resulted in gains of 2.1 % and 0.9 % for small targets, specifically “wearing a helmet (helmet)" and “wearing a helmet (helmet)," respectively, and gave 1.7 % and 1.5 % accuracy boosts to “wearing other clothes (other_clothes)" and “wearing reflective clothing (reflective_clothes)." Model accuracy improved smoothly by 0.2 % for “not wearing a helmet (head)," 0.5 % for “wearing a helmet (helmet)," and 0.5 % for “wearing reflective clothing (reflective clothes)" with the Attention Mechanism module. Performance did not improve for “wearing other clothes,” possibly due to model overfitting. The BiFPN feature fusion network module improved accuracies of “not wearing a helmet (head)," “wearing a helmet (helmet)," and “wearing other clothes (other clothes)" by 1.0 %, 0.8 %, and 2.1 %, respectively. The accuracy of “wearing reflective clothes (reflective clothes)" remained unchanged. The bidirectional flow of feature information facilitates multi-level information interaction and better integrates and utilizes features at different scales. In summary, the P2 detection header significantly enhances overall category performance. Adding the Attention Mechanism module and BiFPN Feature Fusion Network module is prone to overfitting for some category training.

### 4.6 Comparative experiments

Relevant comparison experiments were performed using the same validation dataset to verify the improved model's effectiveness, and results were compared to current mainstream target detection schemes. Table 5 compares the detection results of different schemes on the self-generated dataset. The algorithm surpasses lightweight models such as YOLOv5s, YOLOv6-S, YOLOv7-tiny, and YOLOv8s in accuracy. Additionally, the trained model is only 14.5 MB. Both two-stage algorithms, Faster R–CNN, and single-stage SSD, have lower accuracy and larger models than YOLOv8-MPEB.

**Table 5:** Performance comparison results with other mainstream algorithms.

Detector	Backbone	Params	mAP@50/%	Weight (MB)
Faster R–CNN	VGG16	41.19	83.5	521.7
SSD	VGG16_reducedfc	24.5	79.3	77.4
YOLOv3-tiny	DarkNet-53	12.13	86.8	23.2
YOLOv5s	CSPDarknet53	9.12	89.2	17.6
YOLOv6-S	EfficientRep	16.31	89.5	31.3
YOLOv7-tiny	DenseNet	**6.03**	86.4	**11.8**
YOLOv8s	CSPDarknet53	11.17	89.7	21.4
YOLOv8-MPEB	MobileNetV3	7.39	**91.9**	14.5

### 4.7 Detection effect analysis

**Fig. 12:**
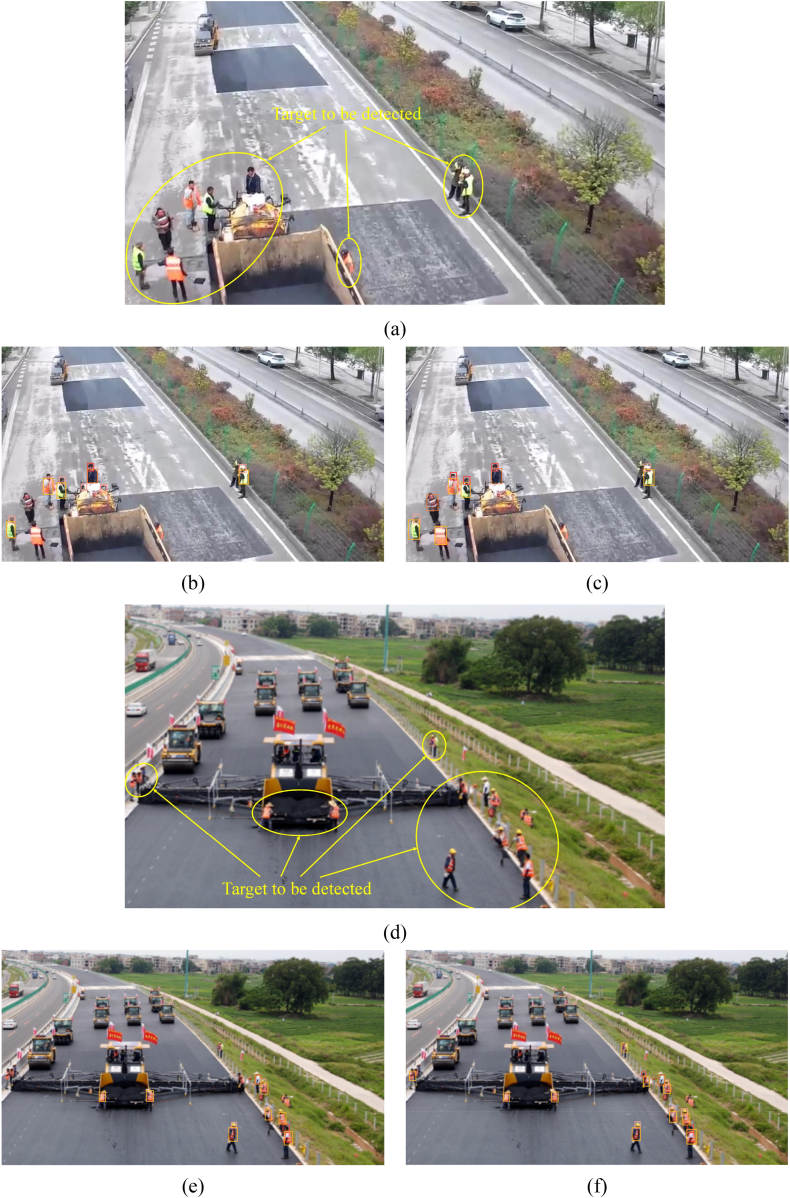

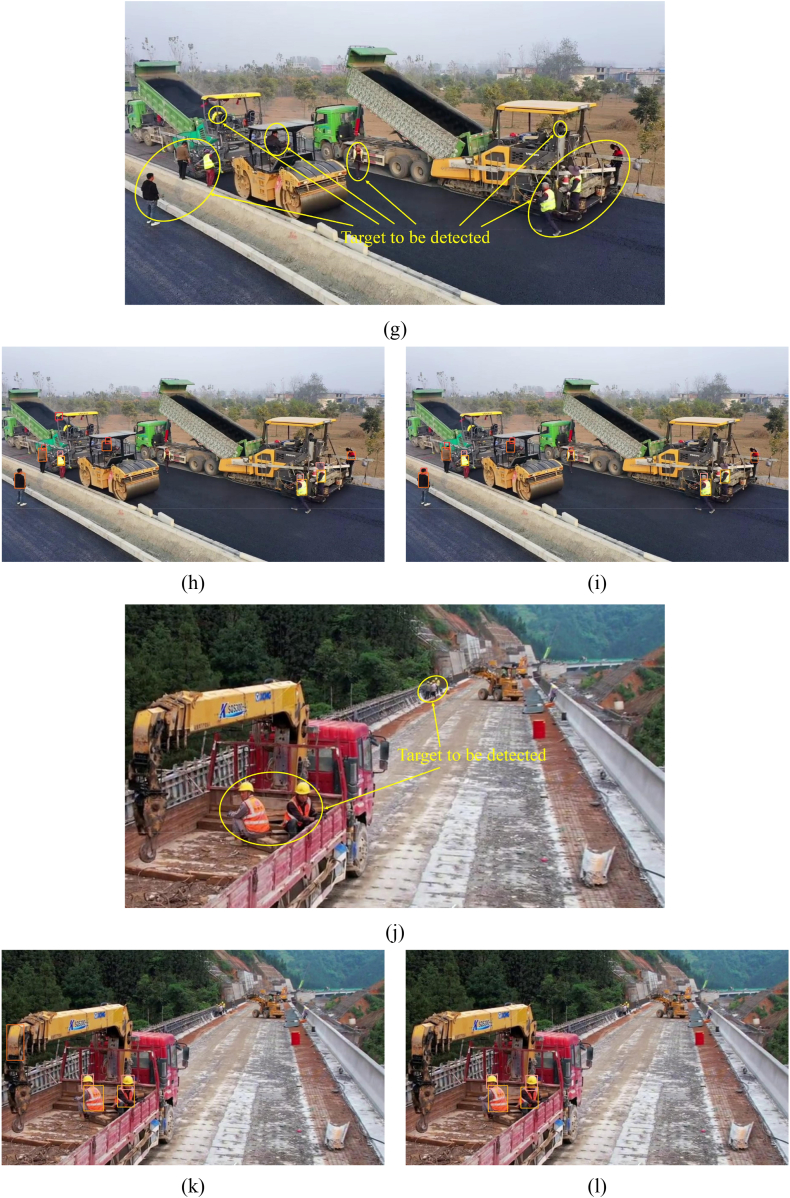
Comparison of detection effect. (a) Road repair site (original photo); (b) Road repair site (inspection effect diagram of YOLOv8s model); (c) Road repair site (detection results of the improved algorithm in this paper); (d) Reconstruction and expansion construction site (original photo); (e) Reconstruction and expansion construction site (inspection effect diagram of YOLOv8s model); (f) Reconstruction and expansion construction site (detection results of the improved algorithm in this paper); (g) Asphalt paving site (original photo); (h) Asphalt paving site (inspection effect diagram of YOLOv8s model); (i) Asphalt paving site (detection results of the improved algorithm in this paper); (j) Bridge construction site (original photo); (k) Bridge construction site (inspection effect diagram of YOLOv8s model); (l) Bridge construction site (detection results of the improved algorithm in this paper).

This paper utilizes YOLOv8s and the improved algorithm to detect road repair sites, reconstruction and expansion construction sites, asphalt pavement paving sites, and bridge construction sites in UAV-captured footage to demonstrate the improved algorithm's detection capabilities. A comparison of the detection results is presented in Fig. 12.

The category selected within the yellow box in the image is “reflective_clothes”, within the orange box is “other_clothes”, within the red box is “head”, and within the pink box is “helmet”. Fig. 12(a), (d), (g), and (j) are original images. Fig. 12(b), (e), (h), and (k) show detection results using the benchmark YOLOv8s algorithm, while Fig. 12(c), (f), (i), and (l) show results using the improved algorithm in this paper. Fig. 12(b) and (c) demonstrate that the proposed algorithm reduces target leakage detection, mainly due to improved small target detection capability. However, aggregated target leakage persists. The issue of missed detection is reduced compared to Fig. 12(e) and (f), but occlusion-related missed detection persists. Fig. 12(h) and (i) show the YOLOv8s algorithm recognizes part of a vehicle as other_clothes and misses two workers; the YOLOv8-MPEB algorithm in this paper does not suffer from these problems but mistakenly recognizes a worker's head as a helmet. Comparing Fig. 12(k) and (l), the YOLOv8s model detects a crane part as other clothes and fails to detect a worker in reflective clothing. However, the algorithm in this paper accurately locates and detects whether the worker is wearing protective gear but fails to detect a tiny distant target.

In summary, the proposed algorithm demonstrates superior performance in multi-scale small-target detection and generalization ability for UAV images compared to YOLOv8s. As demonstrated in this paper, the improved algorithm effectively reduces leakage and false detection in UAV images. However, challenges still need to be solved in detecting tiny, aggregated, and similar targets, resulting in missed or false detections.

## 5 Conclusion

To detect workers wearing protective equipment during road reconstruction and repair, we propose a new system using UAVs and an improved YOLOv8 small target detection algorithm for UAV images. Replacing the backbone network with MobileNetV3 reduces model parameters, computational effort, and size. Adding a small target detection layer and a p2 detection head improves the network's ability to detect small targets. Introducing the C2f module with the EMA attention mechanism reduces target leakage and false positives. Replacing the Neck section with BiFPN, a bidirectional feature pyramid network, enhances the model's generalization ability and improves the detection accuracy of small targets. After numerous experiments on our homemade helmet and reflective clothing dataset, the improved algorithm shows a 2.2 % higher average accuracy for detecting helmet and reflective clothing wear compared to YOLOv8s, with 34 % fewer parameters and a 32 % smaller model size. It meets real-time and accuracy requirements.

The algorithm described in this paper achieves superior results in detecting workers wearing helmets and reflective clothing. It meets requirements for detecting helmet and reflective clothing usage even in complex scenes and changing external factors. However, leakage detection and misdetection of similar categories with dense small targets still occur. There is scope for improving small target detection accuracy. Future work will optimize the multiscale feature pyramid strategy and localization loss function to improve algorithm accuracy and model performance in scenarios with small target aggregations.

## Data availability statement

Data associated with this study has been deposited at https://github.com/a15933312309/Dataset.git.

## Consent for publication

All authors have given consent for publication.

## Funding

This research received no funding.

**Table:** Abbreviations

AP	Average precision
BCE	Binary Cross Entropy
BiFPN	Bidirectional feature pyramid network
C2f	Convolution to feature
C3	Concentrated-Comprehensive Convolution
CA	Coordinate attention
CAM	Channel attention module
CBAM	Convolutional Block Attention Module
CIoU Loss	Complete Intersection over Union Loss
CSPDarknet53	Cross Stage Partial Darknet53
DeepSORT	Deep Simple Online and Realtime Tracking
DFL	Distribution focal loss
EIoU	Expected Intersection over Union
ELAN	Efficient Layer Aggregation Network
EMA	Efficient Multi-scale Attention
FEM	Feature enhancement module
FFNB	Focal FasterNet block
FPN	Feature pyramid network
GFLOPs	Giga floating-point operations per second
mAP	mean Average Precision
MCSA	Multiscale channel-space attention
NAS	Network architecture search
PAN	Path aggregation network
PSA	Pyramid Split Attention
PSAM	Pyramid self-attention module
RCNN	Region-based Convolution Neural Network
ReLU	Rectified Linear Unit
SC_SA	Self-calibrating shuffle attention
SE	Squeeze-and-Excitation
SPIE	International Society for Optical Engineering
SPP	Spatial pyramid pooling
SPPF	Spatial pyramid pooling with features
SSD	Single Shot Multibox Detector
ULSAM	Ultra-Lightweight Quantum Spatial Attention Mechanism
UAV	Unmanned Aerial Vehicle
YOLO	You Only Look Once

## CRediT authorship contribution statement

**Wenyuan Xu:** Supervision, Resources, Data curation, Conceptualization. **Chuang Cui:** Writing – original draft, Validation, Software, Formal analysis. **Yongcheng Ji:** Resources, Formal analysis. **Xiang Li:** Investigation. **Shuai Li:** Formal analysis.

## Declaration of competing interest

The authors declare that they have no known competing financial interests or personal relationships that could have appeared to influence the work reported in this paper.
